# The promoter methylomes of monochorionic twin placentas reveal intrauterine growth restriction-specific variations in the methylation patterns

**DOI:** 10.1038/srep20181

**Published:** 2016-02-02

**Authors:** Zhiming He, Hanlin Lu, Huijuan Luo, Fei Gao, Tong Wang, Yu Gao, Qun Fang, Junwen Wang

**Affiliations:** 1 Foetal Medicine Centre, Department of Obstetrics and Gynaecology, The First Affiliated Hospital of Sun Yat-Sen University, Guangzhou, Guangdong 510080, China; 2 Science & Technology Department, BGI-Shenzhen, No.11, Bei Shan Industrial Zone, Yantian District, Shenzhen 518083, China; 3 Department of Obstetrics and Gynaecology, The Sixth Affiliated Hospital of Sun Yat-Sen University, Guangzhou, Guangdong 510655, China

## Abstract

Intrauterine growth restriction (IUGR) affects the foetus and has a number of pathological consequences throughout life. Recent work has indicated that variations in DNA methylation might cause placental dysfunction, which may be associated with adverse pregnancy complications. Here, we investigated the promoter methylomes of placental shares from seven monochorionic (MC) twins with selective intrauterine growth restriction (sIUGR) using the healthy twin as an ideal control. Our work demonstrated that the IUGR placental shares harboured a distinct DNA hypomethylation pattern and that the methylation variations preferentially occurred in CpG island shores or non-CpG island promoters. The differentially methylated promoters could significantly separate the IUGR placental shares from the healthy ones. Ultra‐performance liquid chromatography/tandem mass spectrometry (UPLC‐MS/MS) further confirmed the genome‐wide DNA hypomethylation and the lower level of hydroxymethylation statuses in the IUGR placental shares. The methylation variations of the *LRAT* and *SLC19A1* promoters, which are involved in vitamin A metabolism and folate transportation, respectively, and the *EFS* promoter were further validated in an additional 12 pairs of MC twins with sIUGR. Although the expressions of *LRAT*, *SLC19A1* and *EFS* were not affected, we still speculated that DNA methylation and hydroxymethylation might serve a functional role during *in utero* foetal development.

Intrauterine growth restriction (IUGR) is one of the most common adverse pregnancy outcomes, affecting up to 7% of pregnancies, with a high risk of perinatal morbidity and mortality^1^,^2^,^3^. Clinically, IUGR is defined as a birth weight below the 10^th^ percentile for the gestational age^4^,^5^, and the babies with IUGR not only suffer from abnormal intrauterine environment and immediate medical problems at birth, but are associated with a significantly increased risk for stroke, type 2 diabetes mellitus, metabolic syndrome, dyslipidemia, cardiovascular and heart disease as well as persistent reduction in growth as adults and may exhibit changes in the pattern of puberty^6^.

Although much is known about the pathological consequences of IUGR, the underlying molecular mechanisms that are responsible for these pathologies are still poorly understood. The placenta is a temporary organ that originates from the trophectoderm layer of the blastocyst and some of the inner cell mass during embryonic development^7^, and has a critical role in regulating the intrauterine environment and nutrient and waste exchange between the maternal and foetal circulation^8^. The differential DNA methylation patterns between the trophectoderm and the inner cell mass during embryonic development play important roles in the placental morphology and physiology^9^. Furthermore, animal models have shown that the environment could affect the placental responses by changing the DNA methylation statuses^10^,^11^. All of these observations shed light on the possibility that variations in the DNA methylation patterns that affect placental development or function might lead to placental insufficiency, foetal growth restriction and death^8^,^12^,^13^.

Recently, DNA imprinting has been widely studied in IUGR placentas^2^,^14^,^15^,^16^,^17^ and the results showed that the loss of *IGF2* imprinting could be involved in the pathophysiology of foetal growth restriction and placental insufficiency^15^. On the other hand, researchers have addressed the DNA methylation patterns of IUGR placental samples by focusing on a limited pool of selected genome-wide CpG dinucleotides, but the observed results were insufficient^8^,^18^,^19^,^20^. We previously demonstrated that full coverage of the cytosine loci was important for evaluating the methylation status within a genomic region, particularly for promoters^21^. The promoter represents an essential regulatory element of gene transcription in the human genome, of which aberrant methylation has been known to be associated with many human diseases^22^. For example, promoter hypermethylation of *Oct4* is associated with down regulation of gene expression and gestational trophoblastic disease^13^. Based on a targeted, regional DNA methylation study, few studies have implied that aberrant promoter methylation might occur in placentas and be associated with IUGR pathogenesis^19^,^20^,^23^,^24^,^25^. However, the variations in entire promoters’ methylation patterns between IUGR and healthy placentas have not been investigated.

Monochorionic (MC) twins harbour the same genetic background, providing an optimal model for investigating epigenomic-mediated molecular mechanisms by using the healthy co-twin as an ideal control. The liquid hybridization capture-based bisulfite sequencing (LHC-BS) is our recently developed method, which employed designed probes to capture the native genomic DNA of interest using a liquid hybridization system, followed by bisulfite treatment and high throughput sequencing to investigate the methylation levels of genomic regions. This strategy was used here to study the promoter methylomes of placental shares from MC twins with selective intrauterine growth restriction (sIUGR)^26^,^27^. The employed probes could continuously interrogate the cytosine methylation statuses from the upstream 2,200 bp to the downstream 500 bp, crossing 91.8% of the transcriptional start sites (TSSs) annotated by the RefSeq gene files (http://genome.ucsc.edu/, hg19), which guaranteed the accuracy of the identification of the differentially methylated regions (DMRs)^21^,^27^.

In this study, seven pairs of MC placentas were employed for whole genomic promoter DNA methylation profiling and some of the candidate differentially methylated promoters were further validated in an additional twelve pairs of samples. Consistent with a previous report^18^, our results indicated that the IUGR-associated placental shares harboured a distinct DNA hypomethylation pattern in promoters and the methylation variations preferentially occurred in CpG island (CGI) shores or non-CGI containing promoters. CGIs are short interspersed DNA sequences with elevated GC content, and preferentially linked with promoters to regulate gene transcriptions^28^. The regional information on the CGIs in this study was referred from the UCSC genome browser database, and the CGI shores were defined as the 2-kb regions that flank the CGI. Mass spectrometry analyses of the seven pairs of MC twins further confirmed that both DNA methylation and hydroxymethylation were significantly lower in the genomic DNA from the IUGR placental shares. Finally, we found that the promoters for *LRAT, SLC19A1*, which are involved in vitamin A metabolism and folate transportation, respectively, and *EFS* were abnormally methylated in the IUGR placental shares. These findings indicated that DNA methylation and hydroxymethylation in placental tissues might serve a functional role in *in utero* foetal development.

## Results

### Sample characteristics and promoter LHC-BS sequencing

In this study, seven pairs of placental shares from MC twins, with one that was diagnosed as sIUGR, were enrolled for LHC-BS sequencing and mass spectrometry analysis. In addition, another twelve pairs of placental samples from MC twins with sIUGR were employed to validate the differentially methylated promoters. The subjects’ clinical characteristics were summarized in Table S1.

The promoter methylomes of the healthy and IUGR placental shares were obtained by LHC-BS sequencing, respectively^26^,^27^. Promoter-targeted LHC-BS could interrogate 1,860,932 CpG sites, of which 138,171 and 5,387 were located in the X and Y chromosomes, respectively, by designing probes covering the upstream 2,200 bp and downstream 500 bp across the transcriptional start sites (TSS) of the Crick strand^27^. A total of 66.6% of the targeted promoters contain CGI(s) and 69.0% of the associated CGI shores are covered as well. Thus, 47.7% of the total CGIs and 35.8% CGI shores in the genome are included in the probes.

From the sequencing results, we generated an average of 5.73 Gb of clean sequencing data for each sample and reached an average of 21.39X coverage depth for the CpGs (Table S2). A total of 86.26% of the generated reads were mapped to at least one genomic position and 74.51% were uniquely mapped. To evaluate the capture efficiency, we found that approximately 97% of the target regions were detected. Furthermore, on average, 91.4% of the designed CpG sites in the Watson strand were detected by at least one depth and 70.3% were detected by at least five depths (Table S2). Although the capture specificity was just approximately 40%, most of the reads that missed the target in the Watson strand were mapped to their complementary strand (the Crick strand) and some reads were flanked on the designed promoter regions. CpG sites with less than 5X sequencing depth and reads mapped to the Crick strand were filtered out, and, as a result, we obtained an average of approximately 1.31 million CpG dinucleotides for each sample for the subsequent analyses (Table S2).

### Global DNA hypomethylation occurred in the placental shares of the IUGR foetus

**Figure 1:**
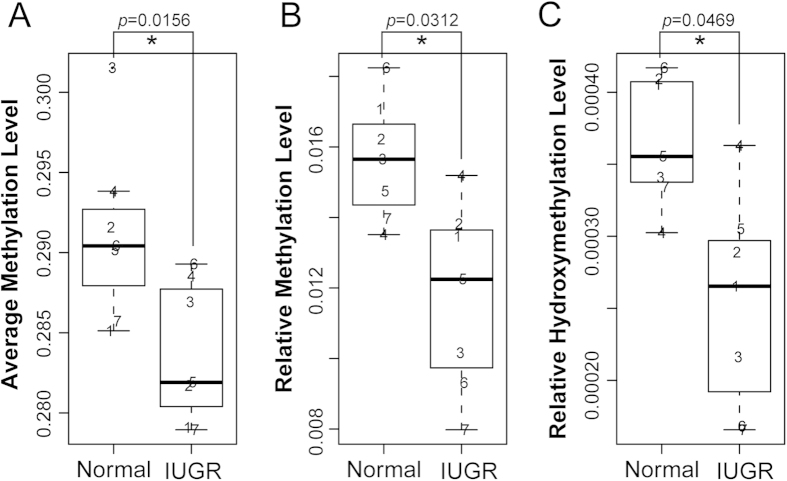
DNA methylation and hydroxymethylation between the placental shares of the IUGR twins and their co-twins. (**A**) The box plot shows the promoter methylation variation between the IUGR placental shares and those of the normal foetuses, as detected by LHC-BS. The box plots show the genomic DNA methylation (**B**) and hydroxymethylation (**C**) variations between the IUGR placental shares and those of their normal co-twins, as detected by UPLC-MS/MS. The numbers were used to differentiate the individuals of the seven MC twins and their locations suggested the average methylation or hydroxymethylation levels. Mann and Whitney test was used for statistical analysis.

First, we evaluated the whole promoter methylation levels of the seven pairs of samples and found that the methylation levels of the IUGR placental shares were significantly lower compared to those of the matched healthy twin (Fig. 1A). To further estimate the distribution of promoter DNA methylation between the IUGR twins’ and co-twins’ placental shares, we divided the promoters into three categories based on the CpG ratio and the length of the CpG-rich regions^29^, namely high-CpG promoters (HCPs), intermediate-CpG promoters (ICPs) and low-CpG promoters (LCPs). As a result, we found that all three kinds of promoters showed reduced DNA methylation in the IUGR placental shares and the variation was more likely to have occurred in the ICP and LCP promoters (Figure S1). In agreement with our result of the tendency for whole promoter DNA hypomethylation in the IUGR placental shares, wide-spread gene enhancer demethylation has been recently reported in placental tissues of early-onset pre-eclampsia^30^, which is a common cause of pre-term birth and foetal growth restriction.

5-hydroxymethylcytosine (5 hmC) is a recently discovered DNA modification in mammalian cells that is catalysed by ten-eleven translocation (TET) dioxygenases using the 5-methylcytosine (5 mC) substrate^31^,^32^. However, bisulfite sequencing could not distinguish 5 hmC from 5 mC^33^. Genomic profiling has revealed that 5 hmC was particularly enriched in the 5 mC-depleted regions, which was thus believed to be involved in the positive DNA demethylation process. 5 hmC is abundant in embryonic stem cells and the brain. However, it is reduced in cancer tissue and is frequently associated with gene and gene regulatory elements^32^,^34^,^35^,^36^, which further indicated that it may serve as more than simply a transient intermediate during the DNA demethylation process and may function in human development and disease.

To further investigate if there is a genome-wide DNA demethylation event in the placental shares of the IUGR foetus and to estimate if 5 hmC plays a functional role in the placental tissue during foetal development *in utero*, we conducted UPLC-MS/MS experiments on the seven MC twin pairs’ placentas. Consistent with our promoter methylome results, we confirmed that the IUGR placental shares were significantly hypomethylated across the genome (Fig. 1B). Interestingly, in contrast with a previous report that 5 hmC was abundant in 5 mC-depleted regions, the UPLC-MS/MS results showed that the 5 hmC levels were also decreased in the IUGR placental shares (Fig. 1C). Pair-wise comparisons between the placental shares of the IUGR and normal foetus within one MC twin further confirmed this conclusion (Figure S2). Based on these findings, we hypothesized that in addition to placental DNA methylation, the hydroxymethylation modification of the genomic DNA might also be important for *in utero* foetal development. Thus, profiling their genome-wide distribution in future studies might further assist us in understanding the involvement of epigenomics in foetal development and associated diseases.

### The promoter methylomes separated the placental shares of the IUGR foetuses from their co-twins

**Figure 2:**
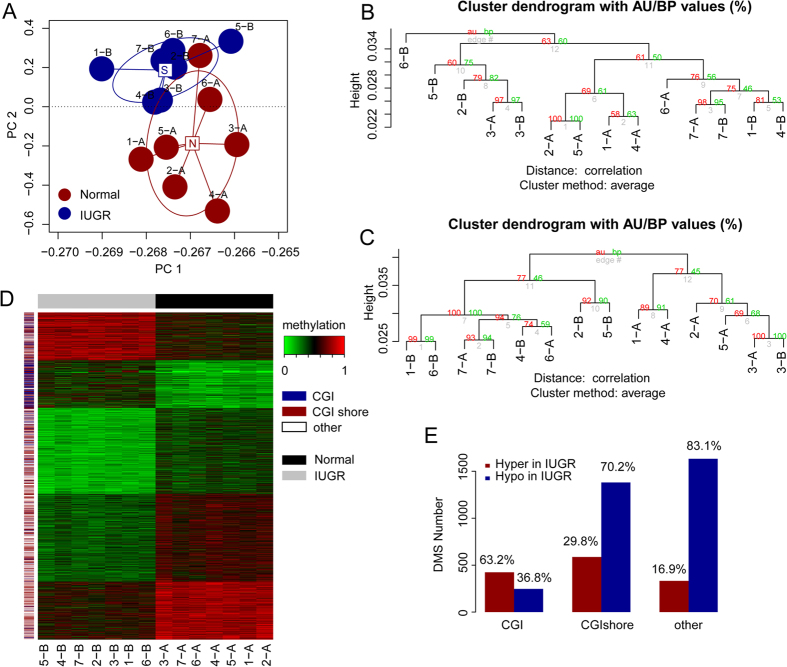
The IUGR placental shares harbour distinct promoter methylomes. (**A**) Principal component analysis (PCA) on the seven pairs of placental shares from the MC twins with sIUGR. Hierarchical clustering of the average DNA methylation levels of the CGI (**B**) and non-CGI promoters (**C**), respectively. (**D**) Clustering of the placental shares based on the differentially methylated sites (DMSs) that were identified according to the criteria of the adjusted *P*-value < 0.05 (Mann and Whitney test) and methylation differences greater than 20%. (**E**) Distribution of the DMSs according to their genomic location. The numbers were used to differentiate the individuals of the seven MC twins; the capital letter “A” indicates the healthy placental shares and the letter “B” indicates the IUGR placental shares.

MC twins are derived from one zygote by splitting into two separate embryos, and they share the same placenta. The DNA methylation statuses were thus expected to be similar between the two individuals or their shared placental tissues^37^. To estimate the DNA methylation variations between the placental shares of the healthy and IUGR foetuses, we selected the commonly covered CpG sites (approximately 1.02 M) in all seven pairs of samples and performed principal component analysis (PCA) based on the methylation level of each site. Interestingly, we found that the IUGR placental tissues were significantly separated from the normal tissues (Fig. 2A). Because the variations may have occurred in the ICP and LCP regions (Figure S1), we further performed a hierarchical clustering analysis on the average methylation levels of the CGI (Fig. 2B) and non-CGI promoters (Fig. 2C), respectively, using the “pvclust” algorithm^38^. In agreement with the PCA results, the methylation levels in both the CGI and non-CGI promoters showed a similar tendency in differentiating the IUGR placental shares from the healthy placental shares, which indicated that the IUGR placental shares harboured a distinct promoter methylation pattern.

To further evaluate the distinct pattern of promoter methylomes between the IUGR and healthy placental shares, we conducted the Mann and Whitney test and identified 4,605 significantly differentially methylated sites (DMSs) with a *P*-value < 0.05 and a methylation difference greater than 20%. Unsupervised clustering of the placentas using DMSs could perfectly discriminate the tissues according to their pathological characteristics (Fig. 2D). In agreement with the finding that the CGI regions are more refractory to methylation variations^37^,^39^, we confirmed that the IUGR placental DNA demethylation mostly occurred in the non-CGI promoters and CGI shores (Fig. 2E). Although a fraction of CpG sites in the CGI promoter displayed variations in methylation, most of the sites became hypermethylated, which is in contrast to the genome-wide demethylation in the IUGR placental shares (Fig. 2E). CGI promoter hypermethylation is frequently associated with transcriptional repression in cancer^22^, which sheds light on its functional roles in IUGR pathogenesis. Taken together, these findings indicated that the IUGR placental shares harboured a distinct promoter methylation characteristic, which might serve as a novel biomarker for monitoring the foetal intrauterine growth status.

### The differentially methylated genes are involved in multiple pathways in the maternal placentas

To minimize the inter-group methylation variations, we performed pair-wise comparisons on the promoter methylation patterns to identify the differentially methylated regions (DMRs). Applying a sliding window strategy with Fisher’s exact test, we identified a total of 2,930 promoters that contained at least one DMR, based on the criteria described in the Methods (Table S3). To estimate the inter-group methylation variations, we further calculated the DMR distributions within each pair of samples. As a result, the data showed that with similar DNA methylation variations, the seven pairs of placentas contained nearly 600 DMRs, respectively (Figure S3).

**Figure 3:**
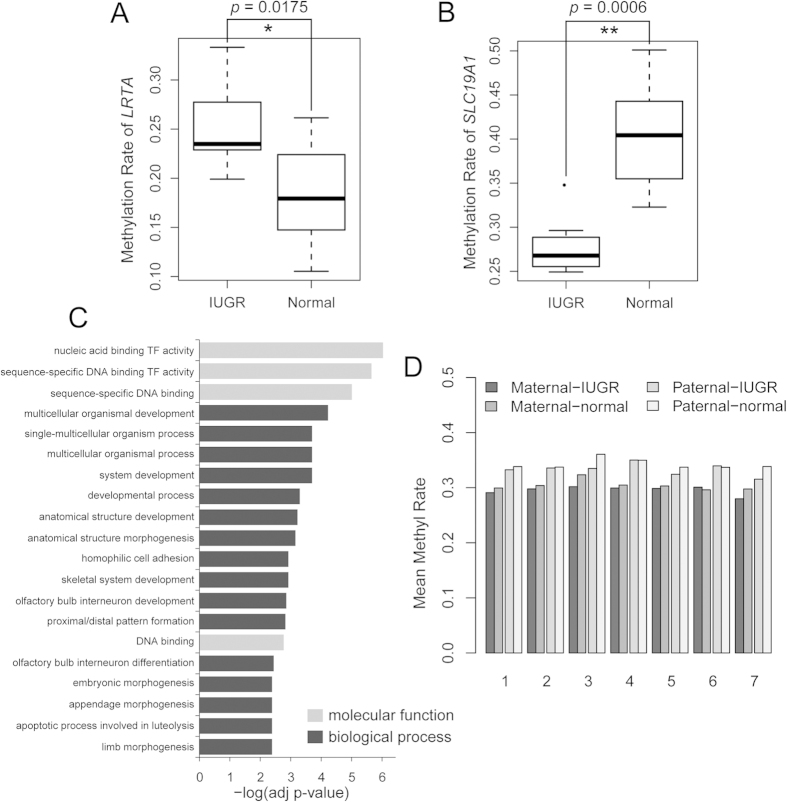
The impact of the differentially methylated promoters of the placental shares on *in utero* foetal growth restriction. The box plots indicated that the *LART* (**A**) and *SLC19A1* (**B**) promoters were significantly differentially methylated in the IUGR placental shares (Mann and Whitney test). (**C**) Gene ontology (GO) analysis of the differentially methylated promoters based on their molecular function and biological processes. (**D**) The methylation variations in the promoters of the maternally and paternally imprinted genes in the IUGR and normal placental shares.

*LOC647288* is a pseudogene that is hypomethylated in all seven IUGR placental shares. Moreover, lecithin retinol acyltransferase (LRAT) is a major enzyme involved in vitamin A metabolism by catalysing the esterification of all-trans-retinol into all-trans-retinyl ester, which regulates various physiological processes. However, it is frequently inhibited by promoter hypermethylation in numerous cancers^40^,^41^,^42^. In this study, we showed that the *LRAT* promoter (GRCh37/Hg19 chr4: 155663541–155664999) was hypermethylated in six IUGR placental shares and the inter-group comparisons displayed a similar result (Fig. 3A), which implied that aberrant DNA methylation might affect *in utero* foetal development through vitamin A metabolism.

To further estimate the functional effects of the DNA methylation variations, we screened 128 promoters that were differentially methylated in no less than three pairs of IUGR placental shares and performed KEGG pathway enrichment and gene ontology (GO) analysis using WebGeStalt^43^. Notably, eight KEGG pathways were significantly enriched with an adjusted *p*-value less than 0.05 (Table S4). In addition to *LRAT*, *SLC19A1*, whose promoter was hypomethylated in four pairs of IUGR placental shares, was also involved in vitamin metabolism pathways. An inter-group comparison confirmed the differential methylation of *SLC19A1* (GRCh37/Hg19 chr21: 46963151–46964484) (Fig. 3B). It has been reported that disturbances in maternal vitamin absorption are connected with restrictions of foetal growth and development and could increase the risk of chronic disease in later life^44^. Furthermore, multiple genes that were involved in the protein digestion and absorption pathway, including *KCNN4, COL12A1* and *PRSS3*, were also aberrantly methylated in most IUGR placentas. Other enriched pathways included Dorso-ventral axis formation, Axon guidance, Maturity onset diabetes of the young, Amyotrophic lateral sclerosis (ALS), Olfactory transduction and Neuroactive ligand-receptor interaction. Gene ontology analysis based on the molecular function and biological processes revealed that the differentially methylated genes were mainly enriched in DNA binding activity and the organismal development process (Fig. 3C, Table S5).

### Comparisons of the promoter methylation of imprinted genes between the IUGR and healthy placental shares

Imprinted genes, which have been thought to function in the regulation of embryonic and placental development as well as in intrauterine foetal growth conditions, are theoretically controlled by DNA methylation in imprinting control regions (ICRs)^2^,^13^. However, their promoter methylation characteristics in IUGR and normal placental tissues have not been studied. Furthermore, it has been reported that some of the allele-specific expressed imprinted genes were even controlled by the promoter DNA methylation statuses, including *Gsα*, *XLsα*, *A/B*, *NESP55* and *GNAS-AS1*^45^. To evaluate if the promoters of imprinted genes are susceptible to methylation changes in response to IUGR, we collected the currently known human imprinted genes from the geneimprint database (www.geneimprint.com) and divided them into two groups based on their paternal allele-specific or maternal allele-specific expression patterns. As a result, we found that the mean methylation levels of the promoters for both maternally and paternally imprinted genes attended to decrease in the IUGR placental shares (Fig. 3D).

### The methylation variations of the *EFS, SLC19A1* and *LRAT* promoters were validated in MC twins with sIUGR

**Figure 4:**
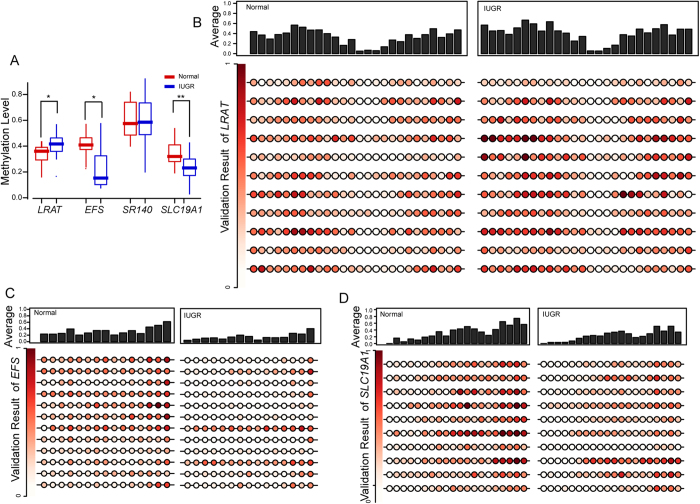
Validation of the variations in promoter DNA methylation. (**A**) The box plots represent the methylation of the *LRAT*, *EFS*, *SR140* and *SLC19A1* promoters in twelve pairs of MC twins with sIUGR. The DNA methylation variations were tested by Mann and Whitney tests (*p-value < 0.05; **p-value < 0.01). The high-resolution methylation variations in the *LRAT* (**B**), *EFS* (**C**) and *SLC19A1* (**D**) genes between the IUGR and normal placental shares were validated by bisulfite genomic sequencing. The circle represents the methylation level of one CpG site on one sample and the depth of the colour represents the methylation variations, as indicated by the rectangle on the left. The upper histogram indicated the associated average CpG methylation levels among the samples.

To further identify the aberrantly methylated genes in the IUGR placental shares, we chose four genes, *LRAT, SLC19A1*, *EFS* and *SR140*, and validated their promoter methylation variations in an additional twelve pairs of MC twins with sIUGR (Table S1). The regional information for validation and the associated primers were summarized in Table S6. Bisulfite genomic sequencing with the multiple clones approach (BGS) was employed, and twelve to twenty subclones for candidate genes were sequenced on a 3730 Genetic Analyzer (Applied Biosystems), respectively. No less than five subclones of a candidate gene were successfully sequenced in one sample. In agreement with the LHC-BS sequencing results, the inter-group comparisons showed that the methylation levels of the *EFS, SLC19A* and *LRAT* promoters were significantly changed in the IUGR placental shares (Fig. 4A). Consistently, pair-wise analyses of the methylation statuses of the CpG sites for each validation region in the IUGR and normal placental shares also showed similar results (Fig. 4B,D).

**Figure 5:**
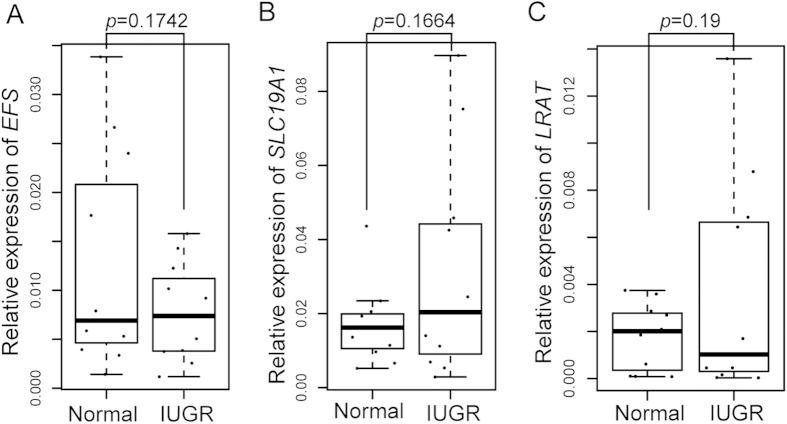
Placental gene expression validation using real time PCR. The box plots showed the 644 expression levels of the *EFS* (**A**), *SLC19A1* (**B**) and *LRAT* (**C**) genes between the IUGR and normal 645 placental shares from eleven MC twins with sIUGR. There was no significant difference in the 28 expression levels of the *EFS* (*p*-value = 0.1742), *SLC19A1* 646 (*p*-value = 0.1664) and *LRAT* genes 647 (*p*-value = 0.19) between the IUGR and normal groups, although there was a negative trend with 648 the methylation levels. The relative expression levels were normalized to the expression of 649 housekeeping gene *GAPDH* and the results were analysed by Mann and Whitney tests.

The protein encoded by *EFS* plays an important role in intracellular signal transduction^46^. Aberrant promoter methylation could significantly inhibit *EFS* expression and is correlated with poor prognosis in cancer^47^. To further explore the correlations between the promoter methylation variations and gene expression in the IUGR placental shares, we concomitantly tested the relative expressions of *EFS, LRAT* and *SLC19A1* in eleven pairs of placental shares using RT-QPCR (Table S1). However, they were not significantly changed by normalizing to the endogenous housekeeping gene of *GAPDH*, based on the collected tissues (Fig. 5). *GAPDH* has been widely used as a standard during testing gene expressions^14^,^15^,^48^, but was reported to be not stably expressed in placental tissues^49^. Thus, we selected another four genes, *SDHA*, *TBP*, *YWHAZ* and *RPL19*, whose expression levels were confirmed to be stable in placental tissues^49^,^50^, as four internal controls to validate the relative expressions. The geometric mean of the four carefully selected housekeeping genes were used for normalization as previously described^51^. However, we still did not find a significant correlation between the promoter methylation variations and gene expression (Figure S4). Further studies to test the relationship between promoter DNA methylation and the gene expression of these candidate genes in homogeneous cells from placentas and further expanding the number of samples in future studies might help us to understand their contributions to IUGR pathogenesis.

## Discussion

Numerous studies have begun to evaluate the correlation between IUGR and the epigenomic modifications in foetal tissues, including the placenta^2^,^8^,^12^,^13^. The placenta plays a central role in foetal development by providing the foetus with nutrients and removing waste from the foetal circulation throughout pregnancy. However, it is subject to different environmental factors, each with the potential to alter the placental epigenomic profiles, which might then contribute to placental dysfunction and adverse pregnancy complications^12^. Many of the current studies were focused on genomic imprinting or identifying the imprinted genes that were speculated to be involved with the occurrence of IUGR^14^,^15^,^16^,^17^. The genome-wide DNA methylation profiles of human placentas were recently investigated using an Illumina Infinium HumanMethylation27 BeadChip array, which confirmed that placental DNA methylation was significantly associated with foetal growth and could function as a marker for the intrauterine environment^18^. This finding provided new insight into epigenomic modifications other than genomic imprinting that are involved in the occurrence of foetal growth restriction *in utero*, as presented here.

We hypothesized that alterations of DNA methylation in promoters, which are recognized as an essential regulatory element for gene expression, might be found in the placentas of neonates and significantly associated with foetal growth *in utero*. LHC-BS is our recently developed method, with single base-pair resolution analysis of the targeted regional DNA methylation status^26^,^27^. Here, we applied the newly developed promoter-targeted LHC-BS approach to investigate the promoter methylome of seven pairs of MC twins’ placentas and further identify the genes that were regulated by promoter methylation but not genomic imprinting throughout the genome. In agreement with Banister’s finding that the IUGR-associated placentas harboured distinct DNA methylation patterns^18^, our result showed that the promoter methylome could significantly distinguish the IUGR placental shares from the normal placental shares (Fig. 2). Furthermore, we found that most of the variations occurred in non-CGI promoters or CGI shores (Fig. 2D,E), which were also preferentially associated with tissue-specific differential methylation regions (T-DMRs) and cancer-specific differential methylation regions (C-DMRs)^52^,^53^.

Previous research demonstrated that the levels of human placental DNA methylation were approximately 2.5–3.5%, which was lower than most somatic tissue (3.5–5%) and human tumours (2–4%)^8^,^54^. However, here, we performed UPLC-MS/MS analyses on the seven pairs of MC twin placentas with sIUGR and showed that the genomic DNA methylation levels in the placental shares from the normal foetuses were lower than 2% (Fig. 1B). This discordance may primarily be due to differences in the techniques used and the regions selected for detection in the previous study. Most interestingly, we simultaneously found that the IUGR placental shares were methylated at much lower levels compared to the normal shares (Fig. 1B). However, the hydroxymethylation level was not increased as anticipated, but was also decreased (Fig. 1C). There is a step-wise genomic demethylation event from fertilization until the morula stage in mammals. After that, DNA methylation is then re-established in a tissue-specific manner during differentiation, which is important for placental morphology and physiology^9^. The inner cell mass, which gives rise to all of the tissues of the adult and some extraembryonic tissues, is hypermethylated, while the trophectoderm, which forms the placenta, becomes hypomethylated^2^,^8^. It is not clear whether the much lower methylation levels in the IUGR-associated placentas were formed during the *de novo* DNA methylation process or affected by environmental factors, but they are very likely to affect placental functions, as revealed by our KEGG pathway enrichment and gene ontology analyses.

One limitation of this study is that the revealed promoter methylome was commixed with the DNA hydroxymethylation modification. Similar to other bisulfite conversion-based methods, LHC-BS could not distinguish 5-hydroxymethylcytosine (5 hmC) from 5-methylcytosine (5 mC)^27^,^33^. Previous studies demonstrated that the 5 hmC levels varied in tissue-specific and cell-type-specific manners^32^,^34^,^36^ and were significantly associated with human disease, including cancer^34^,^35^. Integrated, single base-pair resolution analyses of the distributions of DNA methylation and hydroxymethylation in IUGR and matched placentas will be needed to fully identify the epigenomic contributions to *in utero* foetal development.

The advent of examining the circulating foetal DNA in the maternal peripheral blood provided an optimal approach for non-invasive prenatal diagnosis. In maternal plasma, 3 to 6% of the cell-free DNA during pregnancy is derived from the foetus, and the placenta is the predominant source^55^. Furthermore, the placenta contains different types of cells in both the trophoblast layer and stromal bed^8^. Placental inhomogeneity increases the difficulties of sample collection and might affect the detection accuracy in related studies. Directly identifying the methylation variations in placental tissues could help us to understand the underlying mechanisms, but is hard to apply to prenatal diagnosis. Therefore, it will be important to test if there are methylation variations in the maternal peripheral blood and validate the identified promoters in the cell-free circulating foetal DNA in future studies for applications in non-invasive prenatal diagnosis and foetal development monitoring.

Although we confirmed the methylation variations in the *EFS, LRAT* and *SLC19A1* promoters by both LHC-BS and GBS approaches, their expression levels were not significantly changed between the IUGR and healthy placental shares. One possible reason for this observation may be related to the stability of the mRNA by post-transcriptional mechanism to control gene expression^25^, and the placental inhomogeneity may also affect the detection result. Furthermore, numerous studies have indicated that variation in DNA methylation may not necessarily relate to variation in gene expression, and their relationship is more complex than anticipated^56^. The reason for this is unclear, but it might be related to multiple additional layers of epigenetic modifications cooperating with DNA methylation to regulate gene expression.

In summary, we explored the promoter methylomes and the genome-wide DNA methylation/hydroxymethylation levels in the placentas of MC twins with sIUGR to study the contribution of placental DNA methylation towards foetal development. We revealed that the placental shares of the IUGR foetuses were significantly hypomethylated in both the promoter and throughout the genome and that the methylation profile of the promoters was significantly connected with the foetal IUGR status. In addition, we found that some of the aberrantly methylated genes were involved in multiple metabolic pathways, such as vitamin/protein digestion and absorption during foetal development. Therefore, further estimations of these variations in a homogeneous type of cells from placental tissue with an expanded sample number might help us to understand the contributions of the placenta to IUGR pathogenesis.

## Materials and Methods

### Collection of placenta samples

We collected the placental tissues of 19 pairs of MC twins with sIUGR between May 2012 and April 2013. The study was performed with the approval of the ethics committee of the First-Affiliated Hospital of Sun Yat-sen University and the ethics committee follows the World Medical Association’s (WMA) Declaration of Helsinki-Ethical Principles for Medical Research Involving Human Subjects. The collection of placental tissues was performed with the patients’ informed consent. The chorionicity was determined using an ultrasound at 11–14 weeks as previously described^57^, and further confirmed by placental examination after delivery. The sIUGR is used to define cases when one twin’s birth weight is less than the 10th percentile for the gestational age, as judged by the previous reference for the MC twins birth weight^4^. The pregnancies with severe maternal complications, TTTS and foetal death were excluded. The sIUGR is classified by the Doppler pattern of umbilical artery diastolic flows of the growth restricted twin^58^. sIUGR type I has a normal diastolic flow. Type II is defined by persistently absent/reverse end-diastolic flow, while type III is defined by the presence of intermittent absent/reverse end-diastolic flow (iAREDF).

The placentas were obtained after vaginal delivery or caesarean section. The placental tissue around the individual insertion region of the umbilical cord was collected within 30 minutes after delivery. The placental tissue was excised from inside the placental lobules, avoiding both the maternal surface and the amniotic membrane. The tissue samples were excised and washed 3 times in sterilized, ice-cold PBS to eliminate any blood. The tissues were placed in freezing tubes, deep frozen in liquid nitrogen overnight, and then transferred to a −80 °C freezer for storage.

### DNA extraction and construction of the LHC-BS libraries

The genomic DNA was extracted using the QIAamp DNA Blood Mini Kit (Qiagen) according to the manufacturer’s instructions. After purification, the concentration and integrity of the isolated genomic DNA were analysed on a Qubit fluorometer using a Quant-iT dsDNA HS Assay kit (Invitrogen) and 1% agarose gel electrophoresis, respectively. The promoter-targeted bisulfite conversion libraries were constructed as previously described^27^, with some modifications. Briefly, 1 μg of the genomic DNA was fragmented by an ultrasonic instrument (Covarias) to a mean size of 250 bp, followed by end-repairing, dA addition to the 3′ end and barcode adapter ligation. Four adapter-ligated samples were equally pooled together for the subsequent hybridization step using our previously designed probes^27^; each sample was 250 ng. The specifically captured DNA ligated with adapters was further treated using an EZ DNA Methylation-Gold Kit (ZYMO), followed by purification using a MiniElute PCR purification Kit (Qiagen). After PCR amplification, the products were purified using a 0.8-fold dilution of AMPure beads (Agencourt), and then quantified using the Bioanalyzer analysis system (Agilent) and real time PCR assay. The qualified libraries were finally analysed using an Illumina HiSeq2000.

### UPLC-MS/MS analysis of the global DNA methylation and hydroxymethylation

One microgram of genomic DNA was incubated at 37 °C for 6 h in 150 μl of digestion buffer (6.7 mM MgCl2, 33 mM NaCl and 6.7 mM Tris-HCl pH = 7.9) containing 2.5 U benzonase nuclease, 3 mU phosphodiesterase I from crotalus adamanteus venom and 2 U alkaline phosphatase, calf intestinal (CIP). A microcon centrifugal filter device with a 3,000 D cut-off membrane was used to the remove protein from the digested DNA samples by centrifuging them at 12,000 rpm for 60 min. The mobile phase consisted of 0.1% formic acid (solvent A) and methanol containing 0.1% formic acid (solvent B). The flow rate was set to 500 μL/min. The enzymatically digested DNA samples (5 μL each) were injected for UPLC-MS/MS analysis with the following LC gradient: 0.0–4.0 min, 0 to 50% solvent B; 4.0–6.0 min, 50% solvent B; 6.0–6.1 min, 50% to 5% solvent B; and 6.1–15 min, 5% solvent B. The separated analytes were detected using a 5500 Qtrap linear ion trap quadrupole mass spectrometer equipped with a Turbo V ion source operated in the ESI mode (AB Sciex) with Analyst software (Version 1.5). The Source and Gas were set as follows: gas 1, nitrogen (45 psi); gas 2, nitrogen (40 psi); ion spray voltage, 5,500 V; ion source temperature, 400 °C; and curtain gas, nitrogen (30 psi). The mass spectrometer was operated in the multi-reaction monitoring mode (MRM).

### Bisulfite genomic sequencing

The PCR primers were designed using the online MethPrimer software (www.urogene.org/methprimer) and were listed in Table S6. Approximately 500 ng of the genomic DNA were subjected to the sodium bisulfite treatment and purification using an EZ DNA Methylation-Gold Kit (ZYMO), and a one-fifth volume of the eluted products was used as the template. PCR amplification was performed with a thermal cycling program of 95 °C for 1 min; 38 cycles of 95 °C for 30 s, 58 °C for 30 s, and 72 °C for 30 s; and then a final 5 min extension at 72 °C. The products were purified using the QIAquick Gel Extraction Kit (Qiagen) and sub-cloned. Twenty colonies for each PCR product were selected for sequencing using the 3730 Genetic Analyzer (Applied Biosystems) to evaluate the methylated cytosine levels.

### RNA isolation and quantitative RT-PCR

RNA isolation and reverse-transcription were performed as previously described^59^. The primers used for real-time PCR were shown in Table S7. Real-time PCR was performed with the SYBR^®^ Premix Ex Taq^TM^ (TaKaRa) on an ABI 7500 Real-Time PCR System using the SYBR Green detection protocol. Briefly, the amplification mixture was 0.5 μM primers, 12.5 μL of SYBR^®^ Premix Ex Taq^TM^, and 2 μL of template cDNAs in a total volume of 25 μL. The samples were amplified with the following program: initial denaturation at 95 °C for 30 sec, followed by 45 cycles of denaturation for 5 s at 95 °C, and annealing/elongation for 20 s at 60 °C. All qPCR reactions were run in triplicate. The qPCR results were exported as cycle thresholds (Ct). The relative level of expression of each gene was normalized to the expression level of the *GAPDH* transcript.

### Computational processing of the LHC-BS data

After removing the adapter sequences and filtering out the low quality reads, the bisulfite sequencing reads were directly aligned to the human reference genome (UCSC hg19) using BSMAP 2.73 with the default parameters^60^, which combines genome hashing and bitwise masking to achieve fast and accurate bisulfite mapping. The ratio of the DNA methylation measurement/level of a specific cytosine was then calculated as the number of reads supporting methylation divided by the total number of reads covering that cytosine. CGIs were defined as regions greater than 200 bp with a GC fraction greater than 0.5 and an observed-to-expected ratio for CpG greater than 0.65, as annotated in the UCSC genome browser. Further explanations of the assessments are detailed in the corresponding illustrations of the figures or tables.

DMRs were identified using a sliding window strategy; commonly covered CpG sites with sequencing depth ≥5X between MC-twin samples were selected for the analysis. Fisher’s exact test was first performed to evaluate the significance of the differences in the methylation levels between two samples. CpG sites with significantly different methylation levels (*P* < 0.05) were identified as the initial DMR loci, and their subsequent sites were gradually merged into the candidate DMRs according to following criteria: first, the distance between two neighbouring CpG sites ≤300 bp; second, all CpG sites in one candidate DMR maintain the same methylation tendency (hyper or hypo); and third, one candidate DMR contains no less than 5 CpG sites. Then, for each of the above candidate DMRs, Fisher’s exact test was performed again to filter out those regions with a *P*-value > 0.05 and a difference in mean methylation levels between the two sample <20%. Finally, the Benjamini-Hochberg procedure (False Discovery Rate, FDR) was used to adjust the *P*-values for multiple comparisons with 0.05 thresholds.

### Data Access

The sequencing and processed data have been deposited in the Gene Expression Omnibus (GEO) with the accession number GSE67377. All of the raw UPLC-MS/MS data were deposited in the figshare database (http://dx.doi.org/10.6084/m9.figshare.1368311).

## Additional Information

**How to cite this article**: He, Z. *et al.* The promoter methylomes of monochorionic twin placentas reveal intrauterine growth restriction-specific variations in the methylation patterns. *Sci. Rep.* **6**, 20181; doi: 10.1038/srep20181 (2016).

## Supplementary Material

Supplementary Information

Supplementary Dataset1

## References

[b1] Garite T. J., Clark R. & Thorp J. A. Intrauterine growth restriction increases morbidity and mortality among premature neonates. Am J Obstet Gynecol 191, 481–487 (2004). 15343225 10.1016/j.ajog.2004.01.036

[b2] Koukoura O., Sifakis S. & Spandidos D. A. DNA methylation in the human placenta and fetal growth (review). Mol Med Rep 5, 883–889 (2012). 22294146 10.3892/mmr.2012.763 PMC3493070

[b3] Romo A., Carceller R. & Tobajas J. Intrauterine growth retardation (IUGR): epidemiology and etiology. Pediatr Endocrinol Rev 6, 332–336 (2009). 19404231

[b4] Ananth C. V., Vintzileos A. M., Shen-Schwarz S., Smulian J. C. & Lai Y. L. Standards of birth weight in twin gestations stratified by placental chorionicity. Obstet Gynecol 91, 917–924 (1998). 9610996 10.1016/s0029-7844(98)00052-0

[b5] Figueras F. & Gratacos E. Update on the diagnosis and classification of fetal growth restriction and proposal of a stage-based management protocol. Fetal Diagn Ther 36, 86–98 (2014). 24457811 10.1159/000357592

[b6] Chernausek S. D. Update: consequences of abnormal fetal growth. J Clin Endocrinol Metab 97, 689–695 (2012). 22238390 10.1210/jc.2011-2741 PMC3319209

[b7] Huppertz B. The anatomy of the normal placenta. J Clin Pathol 61, 1296–1302 (2008). 18755720 10.1136/jcp.2008.055277

[b8] Novakovic B. & Saffery R. DNA methylation profiling highlights the unique nature of the human placental epigenome. Epigenomics 2, 627–638 (2010). 22122048 10.2217/epi.10.45

[b9] Nelissen E. C., van Montfoort A. P., Dumoulin J. C. & Evers J. L. Epigenetics and the placenta. Hum Reprod Update 17, 397–417 (2011). 20959349 10.1093/humupd/dmq052

[b10] Chen P. Y. *et al.* Intrauterine calorie restriction affects placental DNA methylation and gene expression. Physiol Genomics 45, 565–576 (2013). 23695884 10.1152/physiolgenomics.00034.2013 PMC3727019

[b11] Serman L. *et al.* The impact of 5-azacytidine on placental weight, glycoprotein pattern and proliferating cell nuclear antigen expression in rat placenta. Placenta 28, 803–811 (2007). 17509679 10.1016/j.placenta.2007.04.001

[b12] Lee S. A. & Ding C. The dysfunctional placenta epigenome: causes and consequences. Epigenomics 4, 561–569 (2012). 23130837 10.2217/epi.12.49

[b13] Maccani M. A. & Marsit C. J. Epigenetics in the placenta. Am J Reprod Immunol 62, 78–89 (2009). 19614624 10.1111/j.1600-0897.2009.00716.x PMC2813777

[b14] Cordeiro A., Neto A. P., Carvalho F., Ramalho C. & Doria S. Relevance of genomic imprinting in intrauterine human growth expression of CDKN1C, H19, IGF2, KCNQ1 and PHLDA2 imprinted genes. J Assist Reprod Genet 31, 1361–1368 (2014). 24986528 10.1007/s10815-014-0278-0 PMC4171407

[b15] Koukoura O. *et al.* Loss of imprinting and aberrant methylation of IGF2 in placentas from pregnancies complicated with fetal growth restriction. Int J Mol Med 28, 481–487 (2011). 21805044 10.3892/ijmm.2011.754

[b16] Lambertini L. *et al.* Differential methylation of imprinted genes in growth-restricted placentas. Reprod Sci 18, 1111–1117 (2011). 21693779 10.1177/1933719111404611

[b17] Turner C. L. *et al.* Methylation analysis of 79 patients with growth restriction reveals novel patterns of methylation change at imprinted loci. Eur J Hum Genet 18, 648–655 (2010). 20104244 10.1038/ejhg.2009.246 PMC2987339

[b18] Banister C. E. *et al.* Infant growth restriction is associated with distinct patterns of DNA methylation in human placentas. Epigenetics 6, 920–927 (2011). 21758004 10.4161/epi.6.7.16079 PMC3154432

[b19] Yuen R. K., Penaherrera M. S., von Dadelszen P., McFadden D. E. & Robinson W. P. DNA methylation profiling of human placentas reveals promoter hypomethylation of multiple genes in early-onset preeclampsia. Eur J Hum Genet 18, 1006–1012 (2010). 20442742 10.1038/ejhg.2010.63 PMC2987406

[b20] Hogg K., Blair J. D., McFadden D. E., von Dadelszen P. & Robinson W. P. Early onset pre-eclampsia is associated with altered DNA methylation of cortisol-signalling and steroidogenic genes in the placenta. PLoS One 8, e62969 (2013). 23667551 10.1371/journal.pone.0062969 PMC3647069

[b21] Wang J. *et al.* Double restriction-enzyme digestion improves the coverage and accuracy of genome-wide CpG methylation profiling by reduced representation bisulfite sequencing. BMC Genomics 14, 11 (2013). 23324053 10.1186/1471-2164-14-11 PMC3570491

[b22] Robertson K. D. DNA methylation and human disease. Nat Rev Genet 6, 597–610 (2005). 16136652 10.1038/nrg1655

[b23] Chelbi S. T. *et al.* Combination of promoter hypomethylation and PDX1 overexpression leads to TBX15 decrease in vascular IUGR placentas. Epigenetics 6, 247–255 (2011). 20962579 10.4161/epi.6.2.13791

[b24] Schrey S. *et al.* Leptin is differentially expressed and epigenetically regulated across monochorionic twin placenta with discordant fetal growth. Mol Hum Reprod 19, 764–772 (2013). 23832168 10.1093/molehr/gat048

[b25] Tzschoppe A. *et al.* DNA methylation of the p66Shc promoter is decreased in placental tissue from women delivering intrauterine growth restricted neonates. Prenat Diagn 33, 484–491 (2013). 23529764 10.1002/pd.4096

[b26] Wang J. *et al.* High resolution profiling of human exon methylation by liquid hybridization capture-based bisulfite sequencing. BMC Genomics 12, 597 (2011). 22151801 10.1186/1471-2164-12-597 PMC3295804

[b27] Gao F. *et al.* Clustering of Cancer Cell Lines Using A Promoter-Targeted Liquid Hybridization Capture-Based Bisulfite Sequencing Approach. Technol Cancer Res Treat 14, 383–394 (2015). 26269607 10.1177/1533034614500416

[b28] Deaton A. M. & Bird A. CpG islands and the regulation of transcription. Genes Dev 25, 1010–1022 (2011). 21576262 10.1101/gad.2037511 PMC3093116

[b29] Weber M. *et al.* Distribution, silencing potential and evolutionary impact of promoter DNA methylation in the human genome. Nat Genet 39, 457–466 (2007). 17334365 10.1038/ng1990

[b30] Blair J. D. *et al.* Widespread DNA hypomethylation at gene enhancer regions in placentas associated with early-onset pre-eclampsia. Mol Hum Reprod 19, 697–708 (2013). 23770704 10.1093/molehr/gat044 PMC3779005

[b31] Tahiliani M. *et al.* Conversion of 5-methylcytosine to 5-hydroxymethylcytosine in mammalian DNA by MLL partner TET1. Science 324, 930–935 (2009). 19372391 10.1126/science.1170116 PMC2715015

[b32] Kriaucionis S. & Heintz N. The nuclear DNA base 5-hydroxymethylcytosine is present in Purkinje neurons and the brain. Science 324, 929–930 (2009). 19372393 10.1126/science.1169786 PMC3263819

[b33] Huang Y. *et al.* The behaviour of 5-hydroxymethylcytosine in bisulfite sequencing. PLoS One 5, e8888 (2010). 20126651 10.1371/journal.pone.0008888 PMC2811190

[b34] Haffner M. C. *et al.* Global 5-hydroxymethylcytosine content is significantly reduced in tissue stem/progenitor cell compartments and in human cancers. Oncotarget 2, 627–637 (2011). 21896958 10.18632/oncotarget.316 PMC3248214

[b35] Lian C. G. *et al.* Loss of 5-hydroxymethylcytosine is an epigenetic hallmark of melanoma. Cell 150, 1135–1146 (2012). 22980977 10.1016/j.cell.2012.07.033 PMC3770275

[b36] Pastor W. A. *et al.* Genome-wide mapping of 5-hydroxymethylcytosine in embryonic stem cells. Nature 473, 394–397 (2011). 21552279 10.1038/nature10102 PMC3124347

[b37] Gordon L. *et al.* Neonatal DNA methylation profile in human twins is specified by a complex interplay between intrauterine environmental and genetic factors, subject to tissue-specific influence. Genome Res 22, 1395–1406 (2012). 22800725 10.1101/gr.136598.111 PMC3409253

[b38] Suzuki R. & Shimodaira H. Pvclust: an R package for assessing the uncertainty in hierarchical clustering. Bioinformatics 22, 1540–1542 (2006). 16595560 10.1093/bioinformatics/btl117

[b39] Kaminsky Z. A. *et al.* DNA methylation profiles in monozygotic and dizygotic twins. Nat Genet 41, 240–245 (2009). 19151718 10.1038/ng.286

[b40] Cheng Y. W. *et al.* High incidence of LRAT promoter hypermethylation in colorectal cancer correlates with tumor stage. Med Oncol 31, 254 (2014). 25260806 10.1007/s12032-014-0254-7 PMC4386722

[b41] Hassel J. C. *et al.* Lecithin retinol acyltransferase as a potential prognostic marker for malignant melanoma. Exp Dermatol 22, 757–759 (2013). 24433184 10.1111/exd.12236

[b42] Ruiz A. *et al.* Molecular and biochemical characterization of lecithin retinol acyltransferase. J Biol Chem, 274, 3834–3841 (1999). 9920938 10.1074/jbc.274.6.3834

[b43] Zhang B., Kirov S. & Snoddy J. WebGestalt: an integrated system for exploring gene sets in various biological contexts. Nucleic Acids Res 33, W741–748 (2005). 15980575 10.1093/nar/gki475 PMC1160236

[b44] Christian P. & Stewart C. P. Maternal micronutrient deficiency, fetal development, and the risk of chronic disease. J Nutr 140, 437–445 (2010). 20071652 10.3945/jn.109.116327

[b45] Grybek V. *et al.* Methylation and transcripts expression at the imprinted GNAS locus in human embryonic and induced pluripotent stem cells and their derivatives. Stem Cell Reports **3**, 432–443 (2014). 10.1016/j.stemcr.2014.07.002 PMC4266011 25241742

[b46] Tchernev V. T. *et al.* The Chediak-Higashi protein interacts with SNARE complex and signal transduction proteins. Mol Med 8, 56–64 (2002). 11984006 PMC2039936

[b47] Neumann L. C. *et al.* EFS shows biallelic methylation in uveal melanoma with poor prognosis as well as tissue-specific methylation. BMC Cancer 11, 380 (2011). 21871071 10.1186/1471-2407-11-380 PMC3175225

[b48] Ke X. *et al.* Intrauterine growth restriction affects hippocampal dual specificity phosphatase 5 gene expression and epigenetic characteristics. Physiol Genomics 43, 1160–1169 (2011). 21828247 10.1152/physiolgenomics.00242.2010 PMC3217330

[b49] Meller M., Vadachkoria S., Luthy D. A. & Williams M. A. Evaluation of housekeeping genes in placental comparative expression studies. Placenta 26, 601–607 (2005). 16085039 10.1016/j.placenta.2004.09.009

[b50] Iglesias-Platas I. *et al.* Altered expression of the imprinted transcription factor PLAGL1 deregulates a network of genes in the human IUGR placenta. Hum Mol Genet 23, 6275–6285 (2014). 24993786 10.1093/hmg/ddu347 PMC4334785

[b51] Vandesompele J. *et al.* Accurate normalization of real-time quantitative RT-PCR data by geometric averaging of multiple internal control genes. Genome Biol 3, RESEARCH0034 (2002). 10.1186/gb-2002-3-7-research0034 PMC126239 12184808

[b52] Doi A. *et al.* Differential methylation of tissue- and cancer-specific CpG island shores distinguishes human induced pluripotent stem cells, embryonic stem cells and fibroblasts. Nat Genet 41, 1350–1353 (2009). 19881528 10.1038/ng.471 PMC2958040

[b53] Irizarry R. A. *et al.* The human colon cancer methylome shows similar hypo- and hypermethylation at conserved tissue-specific CpG island shores. Nat Genet 41, 178–186 (2009). 19151715 10.1038/ng.298 PMC2729128

[b54] Gama-Sosa M. A., Wang R. Y., Kuo K. C., Gehrke C. W. & Ehrlich M. The 5-methylcytosine content of highly repeated sequences in human DNA. Nucleic Acids Res 11, 3087–3095 (1983). 6856456 10.1093/nar/11.10.3087 PMC325950

[b55] Lo Y. M. & Chiu R. W. Prenatal diagnosis: progress through plasma nucleic acids. Nat Rev Genet 8, 71–77 (2007). 17146468 10.1038/nrg1982

[b56] Lam L. L. *et al.* Factors underlying variable DNA methylation in a human community cohort. Proc Natl Acad Sci USA 109 Suppl 2, 17253–17260 (2012). 23045638 10.1073/pnas.1121249109 PMC3477380

[b57] Sepulveda W., Sebire N. J., Hughes K., Odibo A. & Nicolaides K. H. The lambda sign at 10–14 weeks of gestation as a predictor of chorionicity in twin pregnancies. Ultrasound Obstet Gynecol 7, 421–423 (1996). 8807758 10.1046/j.1469-0705.1996.07060421.x

[b58] Gratacos E. *et al.* A classification system for selective intrauterine growth restriction in monochorionic pregnancies according to umbilical artery Doppler flow in the smaller twin. Ultrasound Obstet Gynecol 30, 28–34 (2007). 17542039 10.1002/uog.4046

[b59] Zhang G. L. *et al.* Discordant HIF1A mRNA levels and oxidative stress in placental shares of monochorionic twins with selective intra-uterine growth restriction. Placenta 36, 297–303 (2015). 25573093 10.1016/j.placenta.2014.12.019

[b60] Xi Y. & Li W. BSMAP: whole genome bisulfite sequence MAPping program. BMC Bioinformatics 10, 232 (2009). 19635165 10.1186/1471-2105-10-232 PMC2724425

