# Supplementary material for: The promoter methylomes of monochorionic twin placentas reveal intrauterine growth restriction-specific variations in the methylation patterns
Source: Sci Rep. 2016 Feb 2;6:20181. doi: 10.1038/srep20181 (PMC4735741; doi:10.1038/srep20181)
Supplement: Supplementary Information [file srep20181-s1.pdf]

The promoter methylomes of monochorionic twin placentas reveal intrauterine growth restriction-specific variations in the methylation patterns

Zhiming He[1,*], Hanlin Lu[2,*], Huijuan Luo[2], Fei Gao[2], Tong Wang[2], Yu Gao[3,4], Qun Fang[1,4], and Junwen Wang[2,4,*]

[1] Foetal Medicine Centre, Department of Obstetrics and Gynaecology, The First Affiliated Hospital of Sun Yat-Sen University, Guangzhou, Guangdong 510080, China

[2] Science & Technology Department, BGI-Shenzhen, No.11, Bei Shan Industrial Zone, Yantian District, Shenzhen 518083, China

[3] Department of Obstetrics and Gynaecology, The Sixth Affiliated Hospital of Sun Yat-Sen University, Guangzhou, Guangdong 510655, China

Supplemental Figure Legends

Figure S1. Promoter methylation differentiation between placental shares of the seven MC twins. Box plots showed methylation variations of high-CpG promoters (HCPs) (A), intermediate-CpG promoters (ICPs) (B) and low-CpG promoters (LCPs) (C) in placental shares between normal (blue) and IUGR (red) fetus. (D) The violin plot shows the promoter methylation variation in three different classes between the IUGR placental and those of the normal's. (HCPs, n=14,404; ICPs, n=6,963 and LCPs, n= 5,181. The Mann and Whitney test)

Figure S2. Whole genome-wide 5-methylcytosine (A) and 5-hydroxymethylcytosine (B) modification levels in placental shares from the seven MC twins with sIUGR detected by UPLC-MS/MS, blue (normal), red (IUGR).

Figure S3. DMRs count of the seven paired samples, respectively.

Figure S4. **Placental gene expression validation using real time PCR.** The box plots showed the expression levels of the *EFS* (A), *SLC19A1* (B) and *LRAT* (C) genes between the IUGR and normal placental shares from eleven MC twins with sIUGR. The relative expression levels were normalized to the expression of the four housekeeping genes, *SDHA, RPL19, TBP and YWHAZ,* and the results were analysed by Mann and Whitney tests.

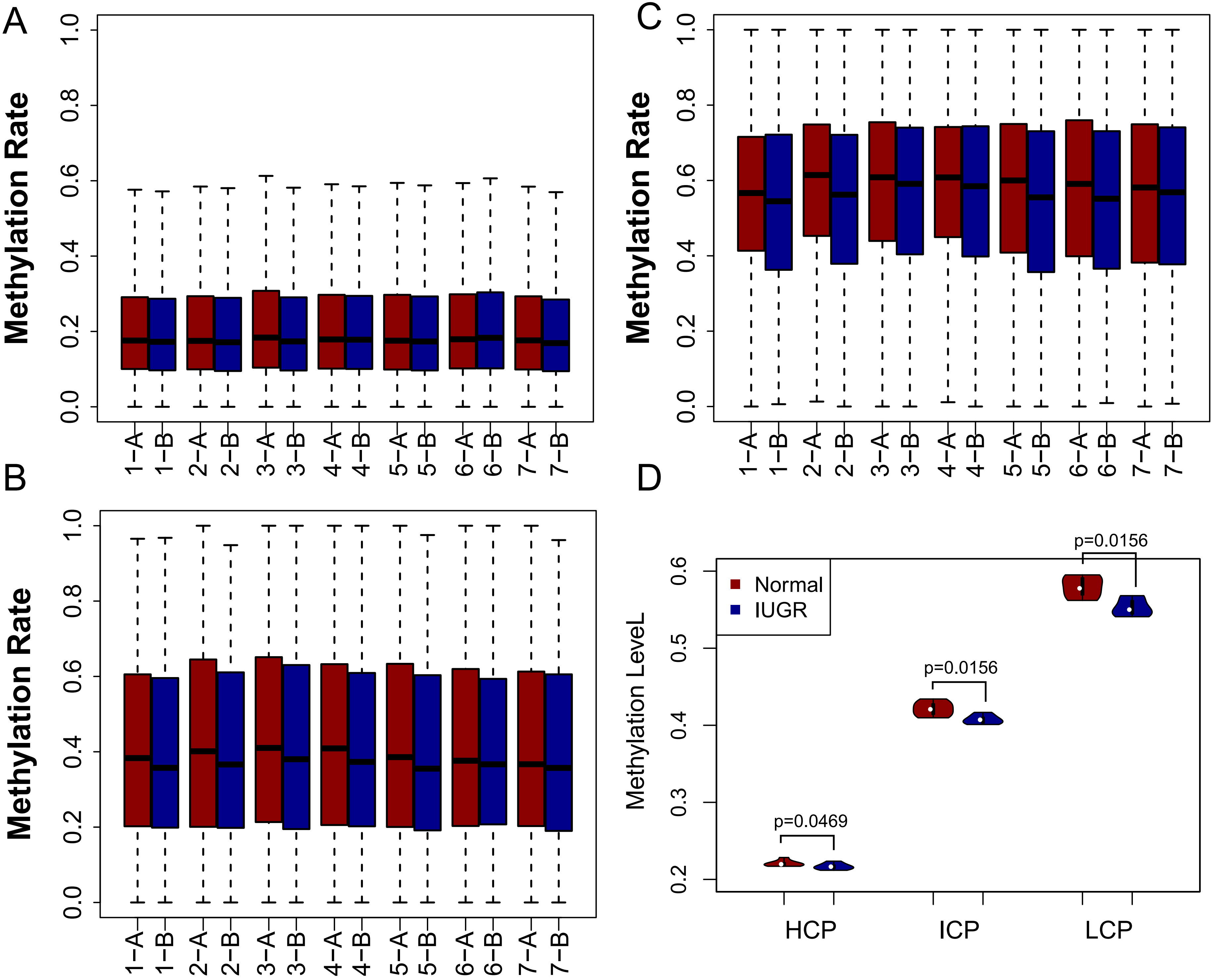
A
B
C
D
Methylation Rate
0.0
0.2
0.4
0.6
0.8
1.0
1−A 1−B 2−A 2−B 3−A 3−B 4−A 4−B 5−A 5−B 6−A 6−B 7−A 7−B
Normal
IUGR
Methylation LeveL
p=0.0469
p=0.0156
p=0.0156
0.2
0.3
0.4
0.5
0.6
HCP
ICP
LCP

Figure S1

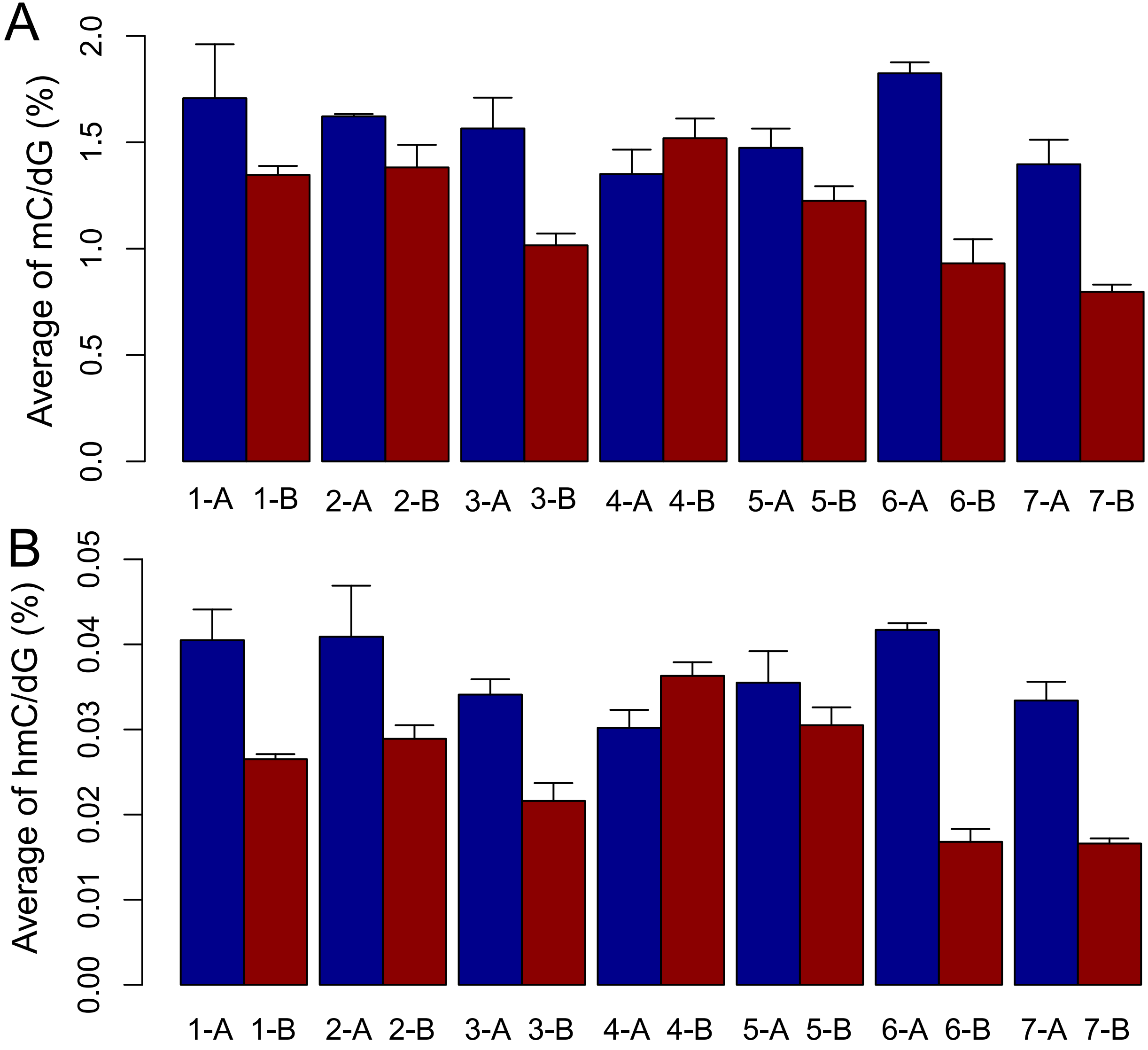
A
B
Average of mC/dG (%)
Average of hmC/dG (%)
0.0
0.5
1.0
1.5
2.0
0.00
0.01
0.02
0.03
0.04
0.05
1-A 1-B 2-A 2-B 3-A 3-B 4-A 4-B 5-A 5-B 6-A 6-B 7-A 7-B
1-A 1-B 2-A 2-B 3-A 3-B 4-A 4-B 5-A 5-B 6-A 6-B 7-A 7-B

Figure S2

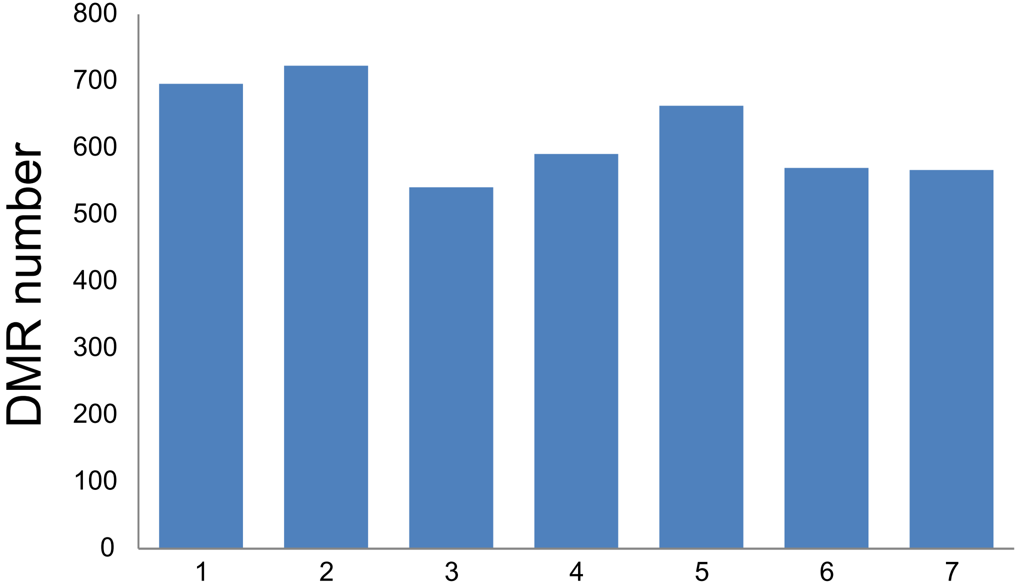

Figure S3

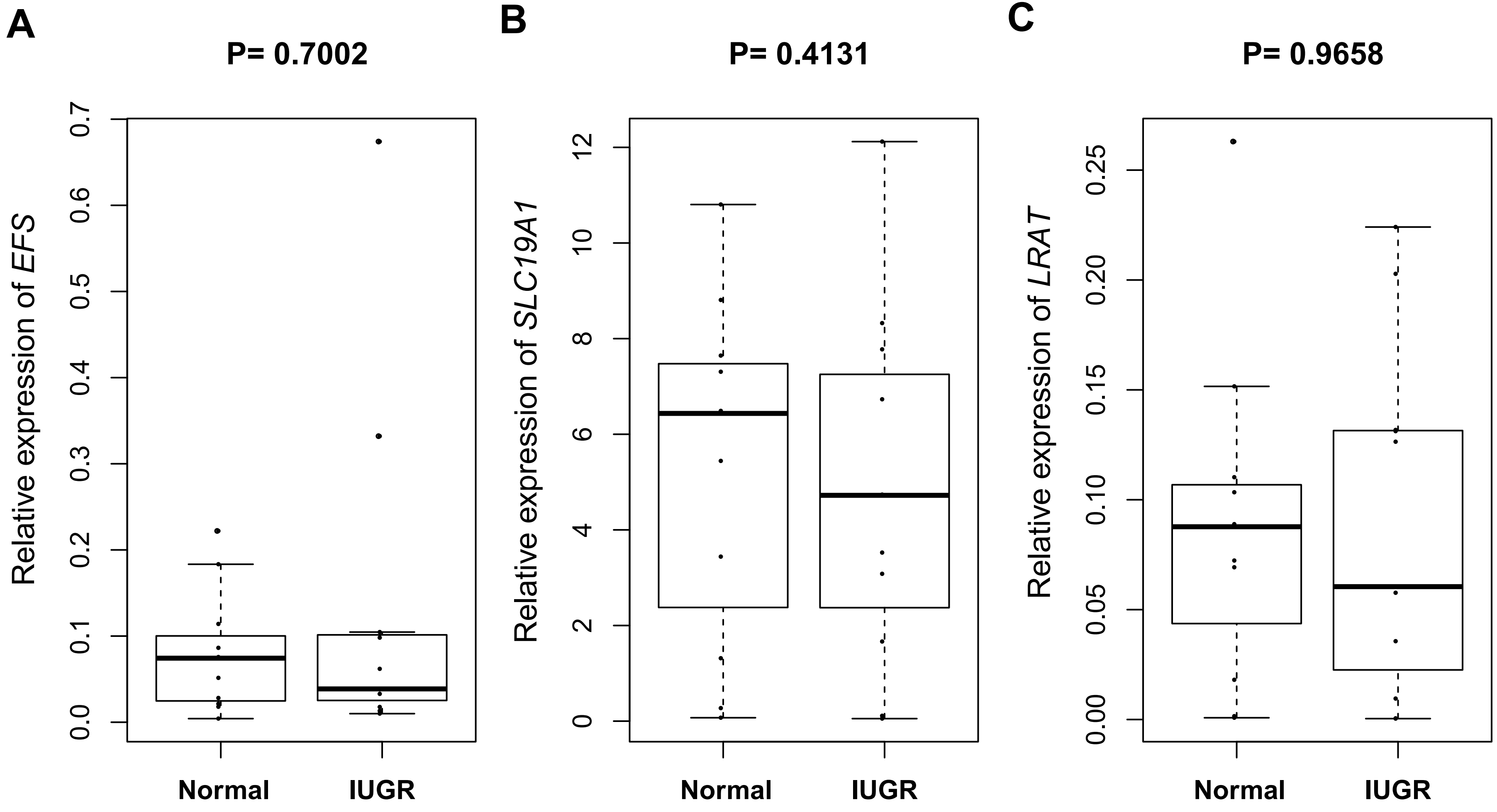
A
P= 0.7002
Relative expression of EFS
0.0
0.1
0.2
0.3
0.4
0.5
0.6
0.7
Normal
IUGR
B
P= 0.4131
Relative expression of SLC19A1
0
2
4
6
8
10
12
Normal
IUGR
C
P= 0.9658
Relative expression of LRAT
0.00
0.05
0.10
0.15
0.20
0.25
Normal
IUGR

Figure S4
